# DEVELOPMENT OF THE TECHNOLOGY INTEGRATION BELIEF INDEX FOR FAMILY CAREGIVERS

**DOI:** 10.1093/geroni/igae098.3547

**Published:** 2024-12-31

**Authors:** Tomoko Wakui, Kentaro Watanabe, Hiroyasu Miwa, Sakiko Itoh

**Affiliations:** Tokyo Metropolitan Institute for Geriatrics and Gerontology, Tokyo, Japan; National Institute of Advanced Industrial Science and Technology, Kashiwa, Chiba, Japan; National Institute of Advanced Industrial Science and Technology, Kashiwa, Chiba, Japan; Tokyo Medical and Dental University, Tokyo, Japan

## Abstract

In highly aged societies, the technology integration into community care settings is promoted. Despite this, real-world adoption faces hurdles because of the common belief that care provided by humans is inherently superior. This gap highlights the absence of specific measures to assess this belief. This study aims to develop the Technology Integration Belief Index for Family Caregivers (TIBI-FC), which evaluates family caregivers’ attitudes towards technology use in long-term care. We conducted a survey with 2,002 family caregivers of older adults living in the community, selected in proportion to the national percentage of older population in need of care. Using SPSS 29.0 and Amos 29, we carried out exploratory and confirmatory factor analyses. Model fit was evaluated using the comparative fit index (CFI) and the root-mean-square error of approximation (RMSEA). The participant group was 42.2% female, with the average age of 60.0 (SD = 10.7). The finalized TIBI-FC, comprising 14 items, was categorized into two dimensions: efficiency and stereotype. The first factor, efficiency, included six items, while the second, stereotype, comprised eight items, demonstrating a robust two-factor structure (CFI =.972, RMSEA =.060). This structure suggests that the TIBI-FC effectively captures the nuanced beliefs about technology integration in care. Importantly, the development of the TIBI-FC not only sheds light on existing challenges but also sets the stage for future strategies to enhance technology acceptance among family caregivers, potentially transforming the landscape of long-term care.

